# First application of an Integrated Biological Response index to assess the ecotoxicological status of honeybees from rural and urban areas

**DOI:** 10.1007/s11356-021-14037-8

**Published:** 2021-04-23

**Authors:** Ilaria Caliani, Tommaso Campani, Barbara Conti, Francesca Cosci, Stefano Bedini, Antonella D’Agostino, Laura Giovanetti, Agata Di Noi, Silvia Casini

**Affiliations:** 1 Department of Physical, Earth and Environmental Sciences, University of Siena, via Mattioli, 4, 53100 Siena, Italy; 2 Department of Agriculture, Food and Environment Entomology, University of Pisa, via del Borghetto, 80, 56124 Pisa, Italy; 3 Department of Management and Quantitative Studies, University of Naples “Parthenope”, via Generale Parisi, 13, 80132 Napoli, Italy; 4 Department of Life Sciences, University of Siena, via Mattioli, 4, 53100 Siena, Italy

**Keywords:** Honeybees, Biomonitoring, Rural areas, Urban pollution, Biomarkers, IBRv2 index

## Abstract

Understanding the effects of environmental contaminants on honeybees is essential to minimize their impacts on these important pollinating insects. The aim of this study was to assess the ecotoxicological status of honeybees in environments undergoing different anthropic pressure: a wood (reference site), an orchard, an agricultural area, and an urban site, using a multi-biomarker approach. To synthetically represent the ecotoxicological status of the honeybees, the responses of the single biomarkers were integrated by the Integrated Biological Response (IBRv2) index. Overall, the strongest alteration of the ecotoxicological status (IBRv2 = 7.52) was detected in the bees from the orchard due to the alteration of metabolic and genotoxicity biomarkers indicating the presence of pesticides, metals, and lipophilic compounds. Honeybees from the cultivated area (IBRv2 = 7.18) revealed an alteration especially in neurotoxicity, metabolic, and genotoxicity biomarkers probably related to the presence of pesticides, especially fungicides. Finally, in the urban area (IBRv2 = 6.60), the biomarker results (GST, lysozyme, and hemocytes) indicated immunosuppression in the honeybees and the effects of the presence of lipophilic compounds and metals in the environment.

## Introduction

The honeybee, *Apis mellifera* L. (Hymenoptera Apidae), is an important pollinator of wild plant species and agricultural crops (Bogdanov et al. 2008; Kurek-Górecka et al. 2020; Simone-Finstrom and Spivak 2010; Thorp 2000). High rates of bees decline have been recorded in the USA (Lee et al. 2015; Kulhanek et al. 2017) and Europe (Brodschneider et al. 2018; Potts et al. 2010), and this threat has led to concerns about sustainable food supply and natural ecosystems health (Cullen et al. 2019). Several authors reported that parasites and diseases, habitat loss, beekeeper management issues, food scarcity, climate change, and contaminant exposure are responsible for the honeybees’ decline (Fairbrother et al. 2014; Goulson et al. 2015; Neumann and Carreck 2010; vanEngelsdorp and Meixner 2010; Williams et al. 2010). Besides, honeybees are exposed to plant protection products (PPPs) such as herbicides, insecticides, fungicides applied to crops (Niell et al. 2017; Porrini et al. 2003), or insecticides used in beekeeping for pest control, mostly against *Varroa destructor* (acaricides) (Calatayud-Vernich et al. 2018; Conti et al. 2020; Mullin et al. 2010). In addition, honeybees can also be in contact with other contaminants present in urban environments, such as PAHs and heavy metals (Caliani et al. 2021; Perugini et al. 2011).

Contaminants reach honeybees during foraging flights, by air flux, and chemical application to the hive (Krupke et al. 2012; Porrini et al. 2003). Inside the hive, contaminants are exchanged among the in-hive bees (DeGrandi-Hoffman and Hagler 2000) so that, within a few hours, the colony is exposed to a cocktail of contaminants (Traynor et al. 2016) that affect not only the individuals but also the colony viability (Calatayud-Vernich et al. 2019). Besides the acute toxic effect of pollutants, the assessment of the sublethal effects of honeybees is important to determine the risks due to environmental pollutants and to minimize their impacts on nontarget pollinating insects.

Biomarkers are a powerful tool very useful to evaluate sublethal effects that occur before irreversible damages to organisms and colonies. In fact, alterations at lower biological levels can be important early warning signals to prevent macroscopic effects at the ecological level. Biomarkers are also ideal tools to investigate the effects of mixtures of contaminants. However, up to now, biomarkers have been mostly evaluated in laboratory studies (Badawy et al. 2015; Badiou et al. 2008; Badiou-Bénéteau et al. 2012; Boily et al. 2013; Caliani et al. 2021; Carvalho et al. 2013; Roat et al. 2017), while few authors used this approach in field studies (Badiou-Bénéteau et al. 2013; Boily et al. 2013; Lupi et al. 2020; Wegener et al. 2016).

Considering the difficulties in analyzing and integrating biomarker responses (Sanchez et al. 2012), an index called Integrated Biological Response (IBRv2), which is based on biomarker deviation from a reference site, has been developed to summarize the biomarker responses (Sanchez et al. 2013). This kind of index is used to quantify the combination of biological effects measured by several biomarkers and to show to which extent each biomarker contributes to the final IBRv2 score (Arrighetti et al. 2019).

Recently, we used the IBRv2 to describe the ecotoxicological status of honeybees assessed by a multi-biomarker approach in the laboratory (Caliani et al. 2021). The present study is aimed to assess the ecotoxicological status of the foraging honeybees in the field by the use of the IBRv2. To do that, foraging honeybees were sampled from hives located in sites undergoing different anthropic pressure, and a wide battery of biochemical and cellular biomarkers (acetylcholinesterase, carboxylesterase, glutathione S-transferase, alkaline phosphatase, lysozyme, hemocytes count, and nuclear abnormalities assay) was assessed to evaluate the potential sublethal effects of multiple contaminants on honeybees.

## Materials and methods

### Sampling sites

Honeybee foragers were sampled from four different areas with different levels of anthropization: an urban site, a cultivated area, an orchard, and a wooded environment.

The beehives used as control were placed in Le Castelline (43.64525 N of latitude, 10.67579 E longitude, and an altitude of 34 m above sea level), a wood area near Pontedera (Pisa, Italy), far from direct sources of urban or intensive agriculture contamination. The urban area (43.29 851 N of latitude, 11.33293 E of longitude, and an altitude of 236 m above sea level) was located 1 km from the center of the city of Siena (Italy) in an anthropized area. In this site, the beehives were positioned just below a beltway. The other beehives were located in an orchard of the Agricultural Faculty of the University of Pisa, at Colignola (Pisa, Italy; 43.72879 N of latitude, 10.46283 E of longitude, and an altitude of 4 m above sea level). The orchard is 3 km far from the Pisa center, and it is characterized by a cultivar collection of different fruiting crops (apple, plum, peach, and grapes) used for experimental and teaching activity and productive purposes. The cultivated area is located near Monteriggioni (Siena, Italy; 43.38740 N of latitude, 11.23109 E of longitude, and an altitude of 252 m above sea level), 10 km from Siena in a land with different crops: cereals, vineyards, olive groves, and vegetables.

### Honeybees

We choose to collect foragers because they represent the individuals primarily exposed in a colony. Sampling was carried out in the summer with the help of beekeepers. About 50 honeybees were randomly collected from three beehives from each sampling site.

### Sample preparation

The honeybees were anesthetized in the laboratory in ice (4°C) for 30 min before being handled. When asleep, the back of the thorax was incised with a scalpel and the hemolymph was collected with a micropipette and used for the hemocytes differential count and nuclear abnormalities (NA) assay. The midgut and the head were immediately frozen and stored at −80°C and then used for the enzymatic biomarkers’ analysis. The head was used to evaluate esterase activity (acetylcholinesterase [AChE] and carboxylesterase [CaE]) whereas glutathione S-transferase (GST), alkaline phosphatase (ALP), and lysozyme (LYS) were evaluated on midgut extract. For the preparation of each extract, tissue samples from 5 specimens were pooled and supernatants obtained according to Caliani et al. (2021).

### Enzyme assays

Acetylcholinesterase (AChE) activity was measured at 412 nm according to the technique described by Ellman et al. (1961) with modifications from Caliani et al. (2021). The carboxylesterase (CaE) enzyme was quantified at 538 nm according to Caliani et al. (2021). GST activity was measured at 340 nm in a medium containing 30-µL extract, 8 mM GSH (reduced glutathione), 8 mM 1-chloro-2,4-dinitrobenzene as the substrate, and 100 mM sodium phosphate pH 7.4. ALP was monitored at 405 nm in a medium containing 100 mM MgCl_2_, 100 mM p-nitrophenyl phosphate as the substrate, and 100 mM Tris–HCl pH 8.5 (Bounias et al. 1996). AChE, CaE, GST, and ALP activities were quantified spectrophotometrically with a Cary UV 60 Agilent spectrophotometer. LYS activity was measured using a turbidity test according to Caliani et al. (2021), and the absorbance was monitored at 450 nm with a Microplate Reader (Model 550, Bio-Rad). Protein concentrations were estimated using the method described by Bradford (1976), with bovine serum albumin as the standard.

### NA assay and hemocytes count

For NA assay, hemolymph from two bees was placed on poly-L-lysine-coated microscope slides and stained with Diff–Quick stain. The NA assay was carried out following the procedure according to Pacheco and Santos (1997) with some modifications. A thousand cells were counted using an immersion light microscope, and different categories of abnormalities (micronuclei, lobed nuclei, segmented, nuclei and kidney-shaped nuclei, apoptotic cells) were attributed (Caliani et al. 2021). Granulocyte and plasmatocytes count were performed following Şapcaliu et al. (2009).

### Statistical analysis

Statistical analysis was carried out with STATA 14 software (StataCorp, 2015). As we considered different biomarkers, the data were first analyzed by comparing the median of the four experimental sites (wood, orchard, cultivated area, and urban area) for each of them. We used both boxplots to explore graphically differences and nonparametric tests. In particular, the significance of the difference between median values was calculated using the Kruskal–Wallis (KW) test and then multiple pairwise comparison tests using the Holm–Sidák adjustment (Sprent and Smeeton, 2016) were performed. Spearman’s rank correlation coefficient was employed to explore the relationship between pairs of biomarkers. The Integrated Biological Response (IBRv2) index (Sanchez et al. 2013) was employed to quantify in a single value the overall degree of contamination of the three experimental sites. The higher the IBRv2 value is, the higher the contamination was.

The detailed procedure to compute IBRv2 is summarized in Caliani et al. (2021) where the same approach was previously used. Spider graphs were used to present the results. The spokes of each spider graph display the value assumed by each biomarker computed as deviation index with respect to its value in the wood.

## Results

**Fig. 1:**
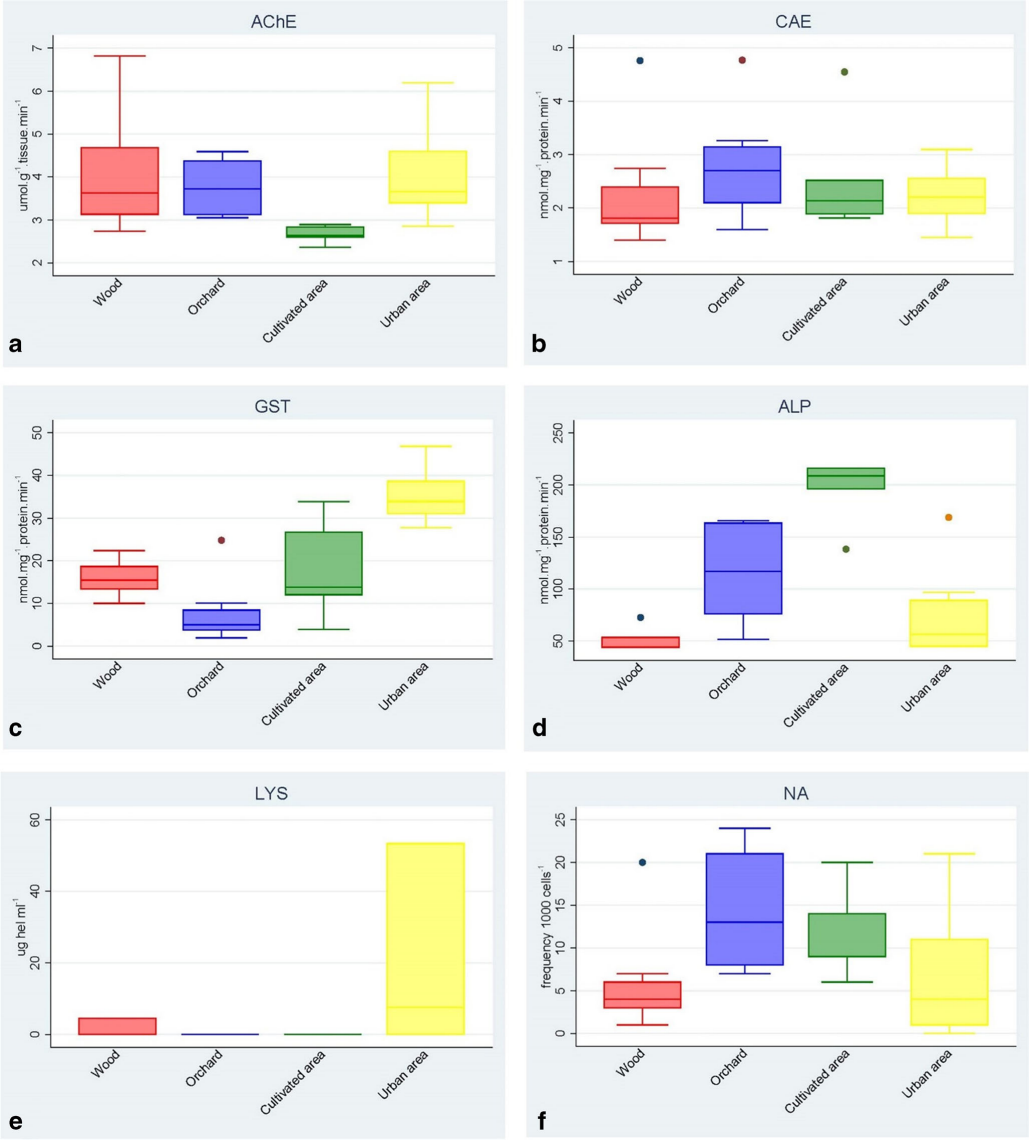
Boxplots of the six biomarkers (activity of acetylcholinesterase, AChE (**a**); carboxylesterase, CaE (**b**); glutathione S-transferase, GST (**c**); alkaline phosphatase, ALP (**d**); lysozyme, LYS (**e**); and nuclear abnormalities (NA) assay (**f**) measured in the forager honeybees, *Apis mellifera*) by the four experimental sites (wood, orchard, cultivated and urban areas)

Foraging honeybees (*n* = 203), collected from four sites with different anthropic pressure, were analyzed using a set of biomarkers: acetylcholinesterase (AChE), carboxylesterase (CaE), glutathione S-transferase (GST), alkaline phosphatase (ALP), lysozyme (LYS), hemocytes count, and nuclear abnormalities (NA) assay. Boxplots of the data are displayed in Fig. 1. Kruskall–Wallis (KW) test highlighted a significant difference between experimental sites, and the results of multiple pairwise comparison tests are reported in Table 1.

**Table 1:** *p* values of the multiple pairwise comparison tests of the six biomarkers

	Cultivated	Urban	Wood
AChE			
Orchard	< 0.01	n.s.	n.s.
Urban	< 0.01	n.s.	n.s.
Wood	< 0.01	n.s.	
CaE			
Orchard	0.051	< 0.05	< 0.01
Urban	n.s.	n.s.	n.s.
Wood	n.s.	n.s.	n.s.
GST			
Orchard	< 0.05	< 0.01	< 0.01
Urban	< 0.01	n.s.	n.s.
Wood	n.s.	< 0.01	n.s.
ALP			
Orchard	< 0.01	n.s.	< 0.01
Urban	< 0.01	n.s.	n.s.
Wood	< 0.01	0.058	n.s.
LYS			
Orchard	n.s.	< 0.01	< 0.05
Urban	< 0.05	n.s.	n.s.
Wood	n.s.	n.s.	n.s.
NA			
Orchard	n.s.	< 0.01	< 0.01
Urban	< 0.01	n.s.	n.s.
Wood	< 0.01	n.s.	n.s.
PLASM			
Orchard	< 0.05	< 0.01	n.s.
Urban	n.s.	n.s.	< 0.01
Wood	n.s.	n.s.	n.s.

Note: n.s. means no significant differences

Data presented in Fig. 1a shows that AChE activity is clearly inhibited in the cultivated area (34% compared to the wood area), with statistically significant differences with respect to all other investigated areas (Table 1). The neural CaE activity (Fig. 1b) increased in all sites with respect to wood, although only the orchard site showed significant statistical induction with respect to wood and urban areas (Table 1). Midgut GST activities (Fig. 1c) were also induced in cultivated and urban areas whereas the orchard appeared statistically inhibited compared to wood and urban areas (Table 1). The ALP activity (Fig. 1d) in midgut did not differ between wood and urban areas, while a strong and statistically significant induction was found in the orchard and cultivated areas (Table 1).

Compared to the control site, lysozyme activity (Fig. 1e) showed similar values in the orchard and cultivated areas and was overactivated in urban areas with values reaching up to 53.39 μg hel ml^−1^.

The hemocyte count showed a decrease of plasmatocytes (PLASM) in the specimens collected in the urban area with statistical differences compared to wood and orchard (Table 1).

Higher values of total nuclear abnormalities (Fig. 1f) were detected in the orchard and cultivated areas with respect to the wood area with a statistical difference (Table 1). Lobed and apoptotic cells were the predominant abnormalities observed in the orchard, with lobed cells showing statistical differences compared to control and urban areas.

Table 2 shows the estimated values of the Spearman’s rank correlation between each couple of biomarkers. This indicator summarizes the strength of association between two variables in a single value between −1 (negative correlation) and +1 (positive correlation). Accordingly, the estimated values suggest a strong positive significant correlation (*p* < 0.01) between LYS and GST and strong negative correlations between LYS and PLASM and LYS and NA (*p* < 0.01).

**Table 2:** Estimated Spearman’s rank correlations between each couple of biomarkers analyzed in honeybees from four different areas.

	NA	AChE	GST	ALP	LYS	CaE	PLASM
NA	1.000						
AChE	−0.345**	1.000					
GST	−0.339**	0.107	1.000				
ALP	0.562**	−0.369**	−0.150	1.000			
LYS	−0.763**	0.204	0.711**	−0.265	1.000		
CaE	0.336**	0.198*	−0.247	−0.089	−0.082	1.000	
PLASM	0.042	0.043	−0.475**	0.428*	−0.793**	0.271*	1.000

Statistically significant correlations are indicated with *p < 0.05 and **p < 0.01.

**Fig. 2:**
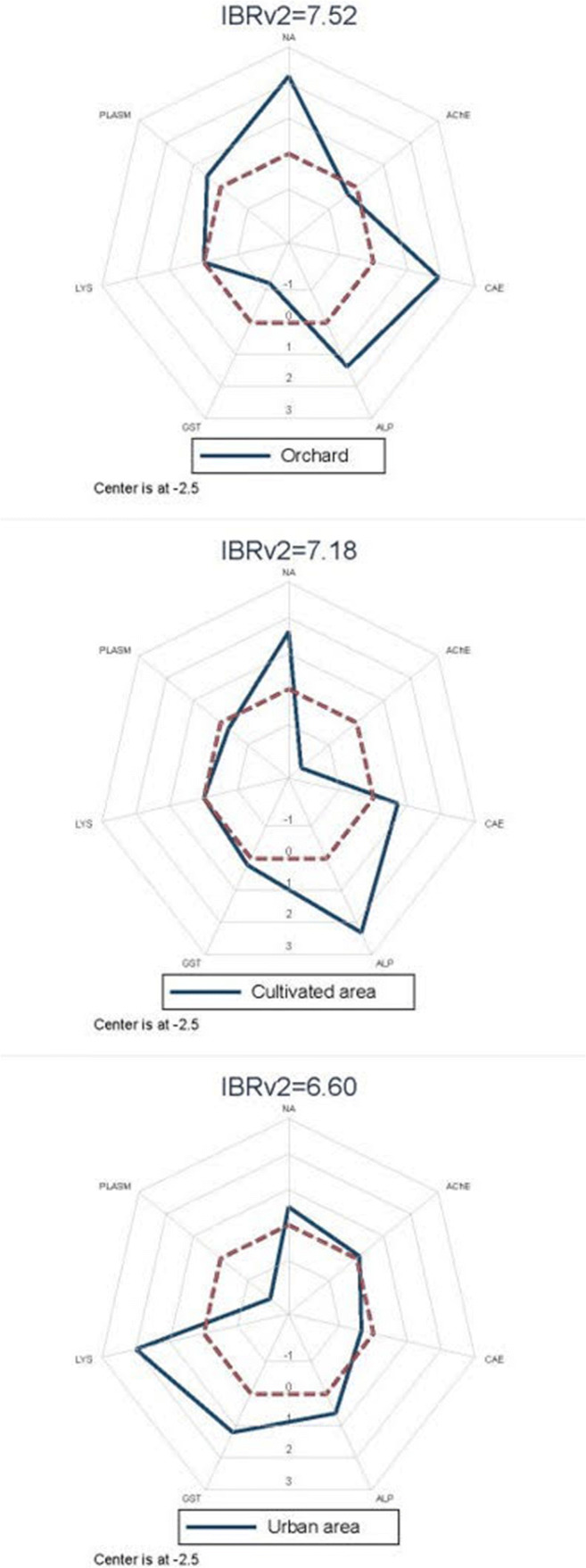
Spider graphs of the Integrated Biological Response (IBRv2)

The results of the Integrated Biological Response (IBRv2) in each area are shown in Fig. 2.

The orchard showed the highest IBRv2 value (7.52), where NA and CaE and ALP values were the most discriminant factors. The cultivated area showed almost the same IBRv2 value (7.18) of the orchard, and NA, AChE, and ALP values were the most relevant responses that explain the IBRv2 indicator. The urban area showed the lowest IBRv2 values (6.60), and the most relevant responses were the LYS, GST, and PLASM.

## Discussion

The combined effects of multiple stressors, rather than a single stress factor, are able to cause adverse effects to and also the death of bee colonies (EFSA 2014; Goulson et al. 2015; Potts et al. 2010). Bees can be exposed to a variety of chemicals of anthropogenic (e.g., PPPs or veterinary drugs) and natural origins (e.g., mycotoxins, flavonoids, plant toxins) (Carnesecchi et al. 2019). The use of a wide battery of biomarkers ensures an accurate diagnosis of exposure and the effects of environmental contaminants, especially when there is a need to monitor different classes of contaminants or mixtures (Roméo et al. 2003). In this study, the responses of selected biomarkers (AChE, CaE, GST, ALP, lysozyme, hemocytes count, and NA assay) were integrated by the IBRv2 index in order to evaluate the impact of environmental pollutants on honeybees and their ecotoxicological status. To the best of our knowledge, this is the first study that evaluates the impact of pollutants from different areas on *A*. *mellifera* by an IBR index.

Overall, in the specimens from the cultivated area, we observed a strong inhibition of AChE, an increase in ALP and GST activities and NA frequency, and a reduction in plasmatocytes. AChE is a biomarker of neurotoxicity (Bandyopadhyay, 1982; Frasco et al. 2005), and a significant reduction in AChE activity has been demonstrated in several studies to be related to the exposure to neurotoxic compounds, such as insecticides and fungicides (Badiou et al. 2008; Badiou-Bénéteau, 2013; Fulton and Key 2001; Lupi et al. 2020; Rabea et al. 2010). The reduced AChE activity found in honeybees from the cultivated site suggests the presence of neurotoxic compounds, while no neural inhibition was found in the other sites. An alteration in neural functions due to pesticides can cause a decrease in foraging activity (Prado et al. 2019) and the general fitness of organisms (Tomé et al. 2020); moreover, pesticides can impair honeybees’ learning and memory, resulting in cognitive disorder that may affect also their dancing communication (Zhang et al. 2020). In this study, we found a significant negative correlation (−0.369, *p* < 0.01) between AChE and ALP, an enzyme involved in adsorption and transport mechanisms (Moss 1992). In particular, in the cultivated area, together with the highest values of ALP activity, we also observed the strongest AChE inhibition. In line with our results, Badiou-Bénéteau et al. (2013) found higher levels of ALP activity and AChE inhibition in a weakly anthropized site in comparison to the control site. Our ALP data are twice higher than the values reported by Caliani et al. (2021) for honeybees exposed to Amistar® Xtra at recommended field usage concentration. This confirms the validity of ALP as a biomarker of honeybees’ exposure to pesticides. Similarly, to ALP activity, NA assay values were found to increase in this area in comparison with the wood area, highlighting genotoxic effects in the specimens analyzed. However, these frequencies were lower (11.07‰) than those measured in our previous work (44.60‰) (Caliani et al. 2021). In the cultivated area, we also observed an increase in GST activity. In general, the induction of GST, an enzyme involved in a bee’s cellular defense processes, reflects exposure to xenobiotics including PPPs. In fact, our findings are in agreement with a previous work where we found that bees exposed in the laboratory to 200 mg/L of Amistar® Xtra, a commercial fungicide composed of azoxystrobin and ciproconazole, induced GST activity (Caliani et al. 2021). Johansen et al. (2007) also observed that 10 mg/L of the fungicide fenpropimorph increased the GST activity in *Mamestra brassicae*, and Han et al. (2014) highlighted an increase in the GST activity in the earthworm *Eisenia fetida* (Savigny, 1826) exposed to azoxystrobin. As already underlined, one of the principal stressors that damage the honeybee’s colonies is pesticides (Desneux et al. 2007; Tosi et al. 2018). Moreover, more than one pesticide is often used by farmers over a growing season. The chronic exposure to pesticides alone or in combination represents a threat for the honeybee populations (Calatayud-Vernich et al. 2016; Gill et al. 2012; Johnson et al. 2013; Tosi et al. 2018; Zhu et al. 2014), altering their physiology through metabolism, immunity, the nervous system, detoxification, and antioxidant defenses (Almasri et al. 2020). Pesticides can reach the hive by contaminated pollen collected by foraging honeybees. During the first days of adult life, the stored pollen is largely used as food; in this way, the colony is chronically exposed to multiple pesticides (Prado et al. 2019). Among pesticides, insecticides (organophosphates, carbamates, pyrethroids, and neonicotinoids) are the most studied for their effects on honeybees (Chalvet-Monfray et al. 1996; Imran et al. 2019; Ma et al. 2019; Williamson and Wright 2013; Wright et al. 2015). Some studies showed that herbicides and fungicides also can affect honeybees’ health status (Balbuena et al. 2015; Decourtye et al. 2005; Devillers 2002; Herbert et al. 2014; Ladurner et al. 2005). Prado et al. (2019) reported that fungicides were the predominant compounds affecting the bee’s energetic metabolism and flight activity, finding them in 60% of the colonies sampled within apiaries. This could support the hypothesis that fungicides, widely used in crops cultivated with cereals, might have a role in the effects observed in this work in the honeybees sampled in the cultivated site.

In the orchard site, CaE activity was significantly induced (19%), while no evidence of AChE inhibition was found. CaEs are hydrolases catalyzing the reactions of aliphatic/aromatic esters of a wide range of xenobiotics (Dauterman, 1985). CaE induction after honeybees’ exposure to several pesticides has been described in several works (Badiou-Bénéteau et al. 2012; Carvalho et al. 2013; Hashimoto et al., 2003; Roat et al. 2017). CaE enzymes are considered to have a double role; in fact, they may be both considered as phase I detoxifying enzymes and also as suicide enzymes that inactivate organophosphate and carbamate insecticides (Dary et al. 1990; Gunning et al. 1997; Stone et al. 2002; Yu et al. 1984). Based on our results, in the orchard, CaE activity probably plays a detoxifying role instead of inactivating neurotoxic compounds. In the orchard site, due to the presence of fruit trees and the consequent large use of PPPs, we expected an induction in GST activities (Caliani et al. 2021; Carvalho et al. 2013); on the contrary, our data showed a decrease in GST activity. Other authors report that GST activity is modulated by insecticides that cause a decrease in its activity (Badiou-Bénéteau et al. 2012; Lupi et al. 2020; Yao et al. 2018). As demonstrated by Deviller et al. (2005), GST is characterized by a greater biological variability than ALP and AChE. In fact, metabolic biomarkers, being directly involved in detoxification processes, are more variable with respect to neural biomarkers since organisms are often exposed to various pollutants. In addition, our results showed a high frequency of nuclear abnormalities, including a 10‰ MN frequency compared to 0‰ MN frequency found in the other sites. These effects could also be due to the presence of lipophilic compounds that are able to inhibit GST activity and at the same time cause genotoxic effects. In agreement with our results, previous studies reported that metals are able to induce the ALP activity (Badiou-Bénéteau et al. 2012; Bounias et al. 1996; Caliani et al. 2021). In summary, the sublethal effects observed in the orchard might be due not only to the presence of pesticides but also to metals or lipophilic compounds potentially present in this site located in the suburban area.

The presence of vehicular traffic, the high population density, and a low presence of crops are the main characteristics of the urban environments. For this reason, a high level of hydrocarbons and metals emitted from fossil fuel combustion of motor vehicles and domestic heating systems are the main causes of pollution in urban environments (Saeedi et al. 2012) and could influence the health of the beehives inhabiting these areas. Exposure to heavy metals, PAHs, and PCBs leads to GST activity induction (Garner and Di Giulio 2012; Papadopoulos et al. 2004; Yu et al. 2012). The strong induction in GST activity (124%) observed in the urban area in this study could confirm the presence of lipophilic compounds and/or heavy metals. Lysozyme and hemocytes are major elements of the honeybees’ immune system responses; in particular, they are involved in the degradation of the bacterial cell and phagocytic activity, respectively (Amdam et al. 2005; Lazarov et al. 2016). In this work, the specimens from the urban area showed an increase in lysozyme activity and granulocyte number and a simultaneous decrease in plasmatocytes count (*ρ* = −0.793; *p* < 0.01); these data could highlight an alteration of immune system function and consequently a loss of immune efficiency. To the best of our knowledge, few papers evaluated immune system alterations related to contamination in honeybees. As reported in a recent review by Di Noi et al. (2021), insecticides are able to affect the immune system by altering the expression of different related genes (Abbo et al. 2017; Christen et al. 2019; Morimoto et al. 2011; Tesovnik et al. 2017; Zhu et al. 2020), hemocytes density, and antimicrobial activity (Brandt et al. 2016). However, as shown in studies performed on different taxa (Mdaini et al. 2019; Wu et al. 2007), chemical compounds and heavy metals could modify bees’ immune system response. The suppression of the immune system in the honeybee may lead to a decrease of the individual performance to population dynamic disorders of the colony (Colin et al. 2004). Overall, the biomarker (GST, lysozyme, and hemocytes) results confirm the presence of contamination characterized by the presence of lipophilic compounds and metals which cause immunosuppression in the honeybees.

The IBR is a useful tool to analyze the effects of environmental pollutants and to determine their impact on organisms (Cao et al. 2019; Liu et al. 2016; Matić et al. 2020). In our study, the orchard showed a higher IBRv2 score among the study areas. The high IBRv2 score obtained for the orchard site indicates a poor honeybees’ ecotoxicological status. In this site, the index was found mainly influenced by CaE activity and NA assay that might indicate the simultaneous presence of contaminants that alter metabolic biomarkers and cause genotoxic effects. The cultivated area showed a quite similar IBRv2 score to the orchards one. However, the index was mainly influenced by different biomarkers, such as AChE, ALP, and NA, that could indicate the presence of PPPs, especially fungicides. The urban area showed a lower IBRv2 score among the study sites. In this case, the IBRv2 score was mainly influenced by GST and LYS activities and by lower plasmatocyte counts. These results might indicate the presence of metals, and lipophilic compounds that are also able to cause immunosuppression in honeybees.

## Conclusions

The widespread decline of honeybees raises concerns about the sustainability of the ecosystem services they provide, including crop pollination and consequently human food supply. To prevent the loss of these important pollinators, the health status of honeybee populations should be assessed before clear signs of distress appear and colony populations collapse. Environmental contaminants and other factors as well as viruses, parasites, pathogens, and lack of genetic diversity might interact and produce synergistic effects on the declining of honeybee populations. For these reasons, as pointed out by the European Food Safety Authority (EFSA), solutions to honeybee decline should implement a holistic risk assessment method (EFSA AHAW 2016; EFSA 2017; Rortais et al. 2017). The ecotoxicological status of forage honeybees sampled in the four areas was assessed by the IBRv2 index. This monitoring approach applied in our study proved to be a powerful and sensitive tool to investigate the sublethal effects of multiple chemicals of anthropogenic and natural origin.

## Data Availability

The datasets used and/or analyzed during the current study are available from the corresponding author on reasonable request.

## References

[CR1] Abbo PM, Kawasaki JK, Hamilton M, Cook SC, DeGrandi-Hoffman G, Li WF, Liu J, Chen YP (2017). Effects of imidacloprid and *Varroa destructor* on survival and health of European honey bees, *Apis mellifera*: survival and health of European honey bees. Insect Science.

[CR2] Almasri H, Tavares DA, Pioz M, Sené D, Tchamitchian S, Cousin M, Brunet J-L, Belzunces LP (2020). Mixtures of an insecticide, a fungicide and a herbicide induce high toxicities and systemic physiological disturbances in winter *Apis mellifera* honey bees. Ecotoxicol Environ Saf.

[CR3] Amdam GV, Aase ALTO, Seehuus S-C, Kim Fondrk M, Norberg K, Hartfelder K (2005). Social reversal of immunosenescence in honey bee workers. Exp Gerontol.

[CR4] Arrighetti F, Landro SM, Lambre ME, Penchaszadeh PE, Teso V (2019). Multiple-biomarker approach in the assessment of the health status of a novel sentinel mussel Brachidontes rodriguezii in a harbor area. Mar Pollut Bull.

[CR5] Badawy MEI, Nasr HM, Rabea EI (2015). Toxicity and biochemical changes in the honey bee *Apis mellifera* exposed to four insecticides under laboratory conditions. Apidologie.

[CR6] Badiou A, Meled M, Belzunces LP (2008). Honeybee *Apis mellifera* acetylcholinesterase—a biomarker to detect deltamethrin exposure. Ecotoxicol Environ Saf.

[CR7] Badiou-Bénéteau A, Carvalho SM, Brunet J-L, Carvalho GA, Buleté A, Giroud B, Belzunces LP (2012). Development of biomarkers of exposure to xenobiotics in the honey bee *Apis mellifera*: application to the systemic insecticide thiamethoxam. Ecotoxicol Environ Saf.

[CR8] Badiou-Bénéteau A, Benneveau A, Géret F, Delatte H, Becker N, Brunet JL, Reynaud B, Belzunces LP (2013). Honeybee biomarkers as promising tools to monitor environmental quality. Environ Int.

[CR9] Balbuena MS, Tison L, Hahn M-L, Greggers U, Menzel R, Farina WM (2015). Effects of sublethal doses of glyphosate on honeybee navigation. J Exp Biol.

[CR10] Bogdanov S, Jurendic T, Sieber R, Gallmann P (2008). Honey for nutrition and health: a review. J Am Coll Nutr.

[CR11] Boily M, Sarrasin B, DeBlois C, Aras P, Chagnon M (2013). Acetylcholinesterase in honey bees (*Apis mellifera*) exposed to neonicotinoids, atrazine and glyphosate: laboratory and field experiments. Environ Sci Pollut Res.

[CR12] Bounias M, Kruk I, Nectoux M, Popeskovic D (1996). Toxicology of cupric salts on honeybees. V. Gluconate and sulfate action on gut alkaline and acid phosphatases. Ecotoxicol Environ Saf.

[CR13] Bradford MM (1976). A rapid and sensitive method for the quantitation of microgram quantities of protein utilizing the principle of protein-dye binding. Anal Biochem.

[CR14] Brandt A, Gorenflo A, Siede R, Meixner M, Büchler R (2016). The neonicotinoids thiacloprid, imidacloprid, and clothianidin affect the immunocompetence of honey bees (*Apis mellifera* L.). J Insect Physiol.

[CR15] Brodschneider R, Gray A, Adjlane N, Ballis A, Brusbardis V, Charrière J-D, Chlebo R, Coffey MF, Dahle B, de Graaf DC, Maja Dražić M, Evans G, Fedoriak M, Forsythe I, Gregorc A, Grzęda U, Hetzroni A, Kauko L, Kristiansen P, Martikkala M, Martín-Hernández R, Aurelio Medina-Flores C, Mutinelli F, Raudmets A, Ryzhikov AV, Simon-Delso N, Stevanovic J, Uzunov A, Vejsnæs F, Wöhl S, Zammit-Mangion M, Danihlík J (2018). Multi-country loss rates of honey bee colonies during winter 2016/2017 from the COLOSS survey. J Apic Res.

[CR16] Calatayud-Vernich P, Calatayud F, Simó E, Picó Y (2016). Efficiency of QuEChERS approach for determining 52 pesticide residues in honey and honey bees. MethodsX.

[CR17] Calatayud-Vernich P, Calatayud F, Simó E, Picó Y (2018). Pesticide residues in honey bees, pollen and beeswax: assessing beehive exposure. Environ Pollut.

[CR18] Calatayud-Vernich P, Calatayud F, Simó E, Pascual Aguilar JA, Picó Y (2019). A two-year monitoring of pesticide hazard in-hive: high honey bee mortality rates during insecticide poisoning episodes in apiaries located near agricultural settings. Chemosphere.

[CR19] Caliani I, Campani T, Conti B, Cosci F, Bedini S, D’Agostino A, Ammendola A, Di Noi A, Gori A, Casini S (2021). Multi-biomarker approach and IBR index to evaluate the effects of different contaminants on the ecotoxicological status of *Apis mellifera*. Ecotoxicol Environ Saf.

[CR20] Cao R, Zhang T, Li X, Zhao Y, Wang Q, Yang D, Qu Y, Liu H, Dong Z, Zhao J (2019). Seawater acidification increases copper toxicity: a multi-biomarker approach with a key marine invertebrate, the Pacific oyster *Crassostrea gigas*. Aquat Toxicol.

[CR21] Carnesecchi E, Svendsen C, Lasagni S, Grech A, Quignot N, Amzal B, Toma C, Tosi S, Rortais A, Cortinas-Abrahantes J, Capri E, Kramer N, Benfenati E, Spurgeon D, Guillot G, Dorne JLCM (2019). Investigating combined toxicity of binary mixtures in bees: meta-analysis of laboratory tests, modelling, mechanistic basis and implications for risk assessment. Environ Int.

[CR22] Carvalho SM, Belzunces LP, Carvalho GA, Brunet J-L, Badiou-Beneteau A (2013). Enzymatic biomarkers as tools to assess environmental quality: a case study of exposure of the honeybee *Apis mellifera* to insecticides: biomarker responses in honeybees exposed to pesticides. Environ Toxicol Chem.

[CR23] Chalvet-Monfray K, Sabatier P, Belzunces LP, Colin ME, Fléché C (1996). Synergy between deltamethrin and prochloraz in bees: modeling approach. Environ Toxicol Chem.

[CR24] Christen V, Vogel MS, Hettich T, Fent K (2019). A vitellogenin antibody in honey bees (*Apis mellifera*): characterization and application as potential biomarker for insecticide exposure. Environ Toxicol Chem.

[CR25] Colin ME, Bonmatin JM, Moineau I, Gaimon C, Brun S, Vermandere JP (2004) A method to quantify and analyze the foraging activity of honey bees: relevance to the sublethal effects induced by systemic insecticides. Arch Environ Contam Toxicol. 47 10.1007/s00244-004-3052-y 10.1007/s00244-004-3052-y 15386133

[CR26] Conti B, Bocchino R, Cosci F, Ascrizzi R, Flamini G, Bedini S (2020). Essential oils against *Varroa destructor* : a soft way to fight the parasitic mite of *Apis mellifera*. J Apic Res.

[CR27] Cullen MG, Thompson LJ, Carolan James C, Stout JC, Stanley DA (2019). Fungicides, herbicides and bees: a systematic review of existing research and methods. PLoS One.

[CR28] Dary O, Georghiou GP, Parsons E, Pasteur N (1990). Microplate adaptation of Gomori’s assay for quantitative determination of general esterase activity in single insects. J Econ Entomol.

[CR29] Decourtye A, Devillers J, Genecque E, Menach KL, Budzinski H, Cluzeau S, Pham-Delègue MH (2005). Comparative sublethal toxicity of nine pesticides on olfactory learning performances of the honeybee *Apis mellifera*. Arch Environ Contam Toxicol.

[CR30] DeGrandi-Hoffman G, Hagler J, 2000. The flow of incoming nectar through a honey bee (*Apis mellifera* L.) colony as revealed by a protein marker 47, 5.

[CR31] Desneux N, Decourtye A, Delpuech J-M (2007). The sublethal effects of pesticides on beneficial arthropods. Annu Rev Entomol.

[CR32] Deviller G, Palluel O, Aliaume C, Asanthi H, Sanchez W, Franco Nava MA, Blancheton J-P, Casellas C (2005). Impact assessment of various rearing systems on fish health using multibiomarker response and metal accumulation. Ecotoxicol Environ Saf.

[CR33] Devillers, J., 2002. Acute toxicity of pesticides to honey bees. In: Devillers, Jame, Pham-Delègue, Min-Hà (Eds), Honey bees: estimating the environmental impact of chemicals. Taylor & Francis, London and New York, pp. 56-66

[CR34] Di Noi A, Casini S, Campani T, Cai G, Caliani I (2021). Review on sublethal effects of environmental contaminants in honey bees (*Apis mellifera*), knowledge gaps and future perspectives. IJERPH.

[CR35] EFSA (2014). Towards an integrated environmental risk assessment of multiple stressors on bees: review of research projects in Europe, knowledge gaps and recommendations. EFS2 12. 10.2903/j.efsa.2014.3594

[CR36] EFSA (2017) Specifications for field data collection contributing to honey bee model corroboration and verification. EFS3 14. 10.2903/sp.efsa.2017.EN-1234

[CR37] EFSA Panel on Animal Health and Welfare (AHAW) (2016) Assessing the health status of managed honeybee colonies (HEALTHY-B): a toolbox to facilitate harmonised data collection. EFS2 14. 10.2903/j.efsa.2016.4578

[CR38] Ellman GL, Courtney KD, Andres V, Featherstone RM (1961). A new and rapid colorimetric determination of acetylcholinesterase activity. Biochem Pharmacol.

[CR39] Fairbrother A, Purdy J, Anderson T, Fell R (2014). Risks of neonicotinoid insecticides to honeybees. Environ Toxicol Chem.

[CR40] Frasco MF, Fournier D, Carvalho F, Guilhermino L (2005). Do metals inhibit acetylcholinesterase (AChE)? Implementation of assay conditions for the use of AChE activity as a biomarker of metal toxicity. Biomarkers.

[CR41] Fulton MH, Key PB (2001). Acetylcholinesterase inhibition in estuarine fish and invertebrates as an indicator of organophosphorus insecticide exposure and effects. Environ Toxicol Chem.

[CR42] Garner LVT, Di Giulio RT (2012). Glutathione transferase pi class 2 (GSTp2) protects against the cardiac deformities caused by exposure to PAHs but not PCB-126 in zebrafish embryos. Comparative Biochemistry and Physiology Part C: Toxicology & Pharmacology.

[CR43] Gill RJ, Ramos-Rodriguez O, Raine NE (2012). Combined pesticide exposure severely affects individual- and colony-level traits in bees. Nature.

[CR44] Goulson D, Nicholls E, Botias C, Rotheray EL (2015). Bee declines driven by combined stress from parasites, pesticides, and lack of flowers. Science.

[CR45] Gunning RV, Moores GD, Devonshire AL (1997). Esterases and fenvalerate resistance in a field population of *Helicoverpa punctigera* (Lepidoptera: Noctuidae) in Australia. Pestic Biochem Physiol.

[CR46] Han Y, Zhu L, Wang J, Wang J, Xie H, Zhang S (2014). Integrated assessment of oxidative stress and DNA damage in earthworms (Eisenia fetida) exposed to azoxystrobin. Ecotoxicol Environ Saf.

[CR47] Herbert LT, Vazquez DE, Arenas A, Farina WM (2014). Effects of field-realistic doses of glyphosate on honeybee appetitive behaviour. J Exp Biol.

[CR48] Imran M, Sheikh UAA, Nasir M, Ghaffar MA, Tamkeen A, Iqbal MA (2019). Do neonicotinoid insecticides impaired olfactory learning behavior in *Apis mellifera*?. Int J Ind Entomol.

[CR49] Johansen NS, Moen LH, Egaas E (2007). Sterol demethylation inhibitor fungicides as disruptors of insect development and inducers of glutathione S-transferase activities in Mamestra brassicae. Comparative Biochemistry and Physiology Part C: Toxicology &amp. Pharmacology.

[CR50] Johnson RM, Dahlgren L, Siegfried BD, Ellis MD (2013). Acaricide, fungicide and drug interactions in honey bees (*Apis mellifera*). PLoS One.

[CR51] Krupke CH, Hunt GJ, Eitzer BD, Andino G, Given K (2012). Multiple routes of pesticide exposure for honey bees living near agricultural fields. PLoS One.

[CR52] Kulhanek K, Steinhauer N, Rennich K, Caron DM, Sagili RR, Pettis JS, Ellis JD, Wilson ME, Wilkes JT, Tarpy DR, Rose R, Lee K, Rangel J, vanEngelsdorp D (2017). A national survey of managed honey bee 2015–2016 annual colony losses in the USA. J Apic Res.

[CR53] Kurek-Górecka A, Górecki M, Rzepecka-Stojko A, Balwierz R, Stojko J (2020). Bee products in dermatology and skin care. Molecules.

[CR54] Ladurner E, Bosch J, Kemp WP, Maini S (2005). Assessing delayed and acute toxicity of five formulated fungicides to *Osmia lignaria* say and *Apis mellifera*. Apidologie.

[CR55] Lazarov S, Zhelyazkova I, Salkova D, Shumkova R, Takova S (2016). Lysozyme levels in haemolymph of worker bees (*Apis mellifera* L.) from bee colonies with different degree of expression of hygienic behaviour. AST.

[CR56] Lee KV, Steinhauer N, Rennich K, Wilson ME, Tarpy DR, Caron DM, Rose R, Delaplane KS, Baylis K, Lengerich EJ, Pettis J, Skinner JA, Wilkes JT, Sagili R, vanEngelsdorp D (2015). A national survey of managed honey bee 2013–2014 annual colony losses in the USA. Apidologie.

[CR57] Liu J, Qu R, Yan L, Wang L, Wang Z (2016). Evaluation of single and joint toxicity of perfluorooctane sulfonate and zinc to Limnodrilus hoffmeisteri: acute toxicity, bioaccumulation and oxidative stress. J Hazard Mater.

[CR58] Lupi D, Tremolada P, Colombo M, Giacchini R, Benocci R, Parenti P, Parolini M, Zambon G, Vighi M (2020). Effects of pesticides and electromagnetic fields on honeybees: a field study using biomarkers. Int J Environ Res.

[CR59] Ma C, Zhang Y, Sun J, Imran M, Yang H, Wu J, Zou Y, Li-Byarlay H, Luo S (2019). Impact of acute oral exposure to thiamethoxam on the homing, flight, learning acquisition and short-term retention of *Apis cerana*. Pest Manag Sci.

[CR60] Matić D, Vlahović M, Ilijin L, Mrdaković M, Grčić A, Filipović A, Perić-Mataruga V (2020). Metallothionein level, non-specific esterases, fitness-related traits and integrated biomarker response (IBR) in larvae of Lymantria dispar L. (Lepidoptera) originating from unpolluted and polluted locations after chronic cadmium treatment. Ecol Indic.

[CR61] Mdaini Z, El Cafsi M, Tremblay R, Pharand P, Gagné J-P (2019). Spatio-temporal variability of biomarker responses and lipid composition of Marphysa sanguinea, Montagu (1813) in the anthropic impacted lagoon of Tunis. Mar Pollut Bull.

[CR62] Morimoto T, Kojima Y, Toki T, Komeda Y, Yoshiyama M, Kimura K, Nirasawa K, Kadowaki T (2011). The habitat disruption induces immune-suppression and oxidative stress in honey bees: habitat disruption of honey bees. Ecology and Evolution.

[CR63] Mullin CA, Frazier M, Frazier JL, Ashcraft S, Simonds R, vanEngelsdorp D, Pettis JS (2010). High levels of miticides and agrochemicals in North American apiaries: implications for honey bee Health. PLoS One.

[CR64] Neumann P, Carreck NL (2010). Honey bee colony losses. J Apic Res.

[CR65] Niell S, Jesús F, Pérez N, Pérez C, Pareja L, Abbate S, Carrasco-Letelier L, Díaz S, Mendoza Y, Cesio V, Heinzen H (2017). Neonicotinoids transference from the field to the hive by honey bees: towards a pesticide residues biomonitor. Sci Total Environ.

[CR66] Pacheco M, Santos MA (1997). Induction of EROD activity and genotoxic effects by polycyclic aromatic hydrocarbons and resin acids on the juvenile Eel (Anguilla anguilla L.). Ecotoxicol Environ Saf.

[CR67] Papadopoulos AI, Polemitou I, Laifi P, Yiangou A, Tananaki C (2004) Glutathione S-transferase in the insect *Apis mellifera* macedonica kinetic characteristics and effect of stress on the expression of GST isoenzymes in the adult worker bee. Comp Biochem Physiol 5 10.1016/j.cca.2004.09.010 15556070

[CR68] Perugini M, Manera M, Grotta L, Abete MC, Tarasco R, Amorena M (2011). Heavy metal (Hg, Cr, Cd, and Pb) contamination in urban areas and wildlife reserves: honeybees as bioindicators. Biol Trace Elem Res.

[CR69] Porrini C, Sabatini AG, Girotti S, Ghini S, Medrzycki P, Grillenzoni F, Bortolotti L, Gattavecchia E, Celli G (2003). Honey bees and bee products as monitors of the environmental contamination. Apiacta.

[CR70] Potts SG, Biesmeijer JC, Kremen C, Neumann P, Schweiger O, Kunin WE (2010). Global pollinator declines: trends, impacts and drivers. Trends Ecol Evol.

[CR71] Prado A, Pioz M, Vidau C, Requier F, Jury M, Crauser D, Brunet J-L, Le Conte Y, Alaux C (2019). Exposure to pollen-bound pesticide mixtures induces longer-lived but less efficient honey bees. Sci Total Environ.

[CR72] Rabea EI, Nasr HM, Badawy MEI (2010). Toxic effect and biochemical study of chlorfluazuron, oxymatrine, and spinosad on honey bees (*Apis mellifera*). Arch Environ Contam Toxicol.

[CR73] Roat TC, Carvalho SM, Palma MS, Malaspina O (2017). Biochemical response of the Africanized honeybee exposed to fipronil: enzymatic assessment of honeybees exposed to fipronil. Environ Toxicol Chem.

[CR74] Roméo M, Mourgaud Y, Geffard A, Gnassia-Barelli M, Amiard JC, Budzinski H (2003). Multimarker approach in transplanted mussels for evaluating water quality in Charentes, France, coast areas exposed to different anthropogenic conditions: biomarker study in transplanted and resident mussels from NW Atlantic. Environ Toxicol.

[CR75] Rortais A, Arnold G, Dorne J-L, More SJ, Sperandio G, Streissl F, Szentes C, Verdonck F (2017). Risk assessment of pesticides and other stressors in bees: principles, data gaps and perspectives from the European Food Safety Authority. Sci Total Environ.

[CR76] Saeedi M, Li LY, Salmanzadeh M (2012). Heavy metals and polycyclic aromatic hydrocarbons: pollution and ecological risk assessment in street dust of Tehran. J Hazard Mater.

[CR77] Sanchez W, Burgeot T, Perceval O (2012). Perspectives from the French workshop on the development and validation of biomarkers and bioassays for the monitoring of aquatic environments. Environ Sci Pollut Res.

[CR78] Sanchez W, Burgeot T, Porcher J-M (2013). A novel “integrated biomarker response” calculation based on reference deviation concept. Environ Sci Pollut Res.

[CR79] Simone-Finstrom M, Spivak M (2010). Propolis and bee health: the natural history and significance of resin use by honey bees. Apidologie.

[CR80] Stone D, Jepson P, Laskowski R (2002). Trends in detoxification enzymes and heavy metal accumulation in ground beetles (Coleoptera: Carabidae) inhabiting a gradient of pollution. Comparative Biochemistry and Physiology Part C: Toxicology & Pharmacology.

[CR81] Tesovnik T, Cizelj I, Zorc M, Čitar M, Božič J, Glavan G, Narat M (2017). Immune related gene expression in worker honey bee (*Apis mellifera* carnica) pupae exposed to neonicotinoid thiamethoxam and Varroa mites (Varroa destructor). PLoS One.

[CR82] Thorp RW (2000). The collection of pollen by bees. Plant Syst Evol.

[CR83] Tomé HVV, Schmehl DR, Wedde AE, Godoy RSM, Ravaiano SV, Guedes RNC, Martins GF, Ellis JD (2020). Frequently encountered pesticides can cause multiple disorders in developing worker honey bees. Environ Pollut.

[CR84] Tosi S, Costa C, Vesco U, Quaglia G, Guido G (2018). A 3-year survey of Italian honey bee-collected pollen reveals widespread contamination by agricultural pesticides. Sci Total Environ.

[CR85] Traynor KS, Pettis JS, Tarpy DR, Mullin CA, Frazier JL, Frazier M, vanEngelsdorp D (2016). In-hive pesticide exposome: assessing risks to migratory honey bees from in-hive pesticide contamination in the Eastern United States. Sci Rep.

[CR86] vanEngelsdorp D, Meixner MD (2010). A historical review of managed honey bee populations in Europe and the United States and the factors that may affect them. J Invertebr Pathol.

[CR87] Wegener J, Ruhnke H, Milchreit K, Kleebaum K, Franke M, Mispagel S, Bischoff G, Kamp G, Bienefeld K (2016). Secondary biomarkers of insecticide-induced stress of honey bee colonies and their relevance for overwintering strength. Ecotoxicol Environ Saf.

[CR88] Williams GR, Tarpy DR, vanEngelsdorp D, Chauzat M-P, Cox-Foster DL, Delaplane KS, Neumann P, Pettis JS, Rogers REL, Shutler D (2010). Colony collapse disorder in context. Bioessays.

[CR89] Williamson SM, Wright GA (2013). Exposure to multiple cholinergic pesticides impairs olfactory learning and memory in honeybees. J Exp Biol.

[CR90] Wright GA, Softley S, Earnshaw H (2015). Low doses of neonicotinoid pesticides in food rewards impair short-term olfactory memory in foraging-age honeybees. Sci Rep.

[CR91] Wu SM, Shih M-J, Ho Y-C (2007). Toxicological stress response and cadmium distribution in hybrid tilapia (Oreochromis sp.) upon cadmium exposure. Comparative Biochemistry and Physiology Part C: Toxicology & Pharmacology.

[CR92] Yao J, Zhu YC, Adamczyk J (2018). Responses of honey bees to lethal and sublethal doses of formulated clothianidin alone and mixtures. J Econ Entomol.

[CR93] Yu SJ, Robinson FA, Nation JL (1984). Detoxication capacity in the honey bee, *Apis mellifera* L. Pestic Biochem Physiol.

[CR94] Yu X, Sun R, Yan H, Guo X, Xu B (2012). Characterization of a sigma class glutathione S-transferase gene in the larvae of the honeybee (*Apis cerana* cerana) on exposure to mercury. Comp Biochem Physiol B: Biochem Mol Biol.

[CR95] Zhang ZY, Li Z, Huang Q, Zhang XW, Ke L, Yan WY, Zhang LZ, Zeng ZJ (2020). Deltamethrin impairs honeybees (*Apis mellifera*) dancing communication. Arch Environ Contam Toxicol.

[CR96] Zhu W, Schmehl DR, Mullin CA, Frazier JL (2014). Four common pesticides, their mixtures and a formulation solvent in the hive environment have high oral toxicity to honey bee larvae. PLoS One.

[CR97] Zhu L, Qi S, Xue X, Niu X, Wu L (2020). Nitenpyram disturbs gut microbiota and influences metabolic homeostasis and immunity in honey bee (*Apis mellifera* L.). Environ Pollut.

